# The distribution and expression of the Bloom's syndrome gene product in normal and neoplastic human cells

**DOI:** 10.1054/bjoc.2001.1874

**Published:** 2001-07

**Authors:** H Turley, L Wu, M Canamero, K C Gatter, I D Hickson

**Affiliations:** Department of Cellular Science University of Oxford, John Radcliffe Hospital, Oxford, OX3 9DS, UK; ICRF Laboratories, Weatherall Institute of Molecular Medicine, University of Oxford, John Radcliffe Hospital, Oxford, OX3 9DS, UK

## Abstract

Bloom's syndrome (BS) is an autosomal recessive disorder associated with a predisposition to cancers of all types. Cells from BS sufferers display extreme genomic instability. The BS gene product, BLM, is a 159 kDa DNA helicase enzyme belonging to the RecQ family. Here, we have analysed the distribution of BLM in normal and tumour tissues from humans using a recently characterized, specific monoclonal antibody. BLM was found to be localized to nuclei in normal lymphoid tissues, but was largely absent from other normal tissues analysed with the exception of the proliferating compartment of certain tissues. In contrast, expression of BLM was observed in a variety of tumours of both lymphoid and epithelial origin. A strong correlation was observed between expression of BLM and the proliferative status of cells, as determined by staining for markers of cell proliferation (PCNA and Ki67). We conclude that BLM is a proliferation marker in normal and neoplastic cells in vivo, and, as a consequence, is expressed at a higher level in tumours than in normal quiescent tissues. © 2001 Cancer Research Campaign http://www.bjcancer.com


*British Journal of Cancer* (2001) **85**(2), 261–265
© 2001 Cancer Research Campaign
doi: 10.1054/ bjoc.2001.1874, available online at http://www.idealibrary.com on IDEAL®

http://www.bjcancer.com

### The distribution and expression of the Bloom's syndrome gene product in normal and neoplastic human cells

**H Turley[1,2], L Wu[2], M Canamero[1], KC Gatter[1] and ID Hickson[2]**

Department of Cellular Science[1] and ICRF Laboratories, Weatherall Institute of Molecular Medicine[2], University of Oxford, John Radcliffe Hospital, Oxford OX3 9DS, UK

**Summary** Bloom's syndrome (BS) is an autosomal recessive disorder associated with a predisposition to cancers of all types. Cells from BS sufferers display extreme genomic instability. The BS gene product, BLM, is a 159 kDa DNA helicase enzyme belonging to the RecQ family. Here, we have analysed the distribution of BLM in normal and tumour tissues from humans using a recently characterized, specific monoclonal antibody. BLM was found to be localized to nuclei in normal lymphoid tissues, but was largely absent from other normal tissues analysed with the exception of the proliferating compartment of certain tissues. In contrast, expression of BLM was observed in a variety of tumours of both lymphoid and epithelial origin. A strong correlation was observed between expression of BLM and the proliferative status of cells, as determined by staining for markers of cell proliferation (PCNA and Ki67). We conclude that BLM is a proliferation marker in normal and neoplastic cells in vivo, and, as a consequence, is expressed at a higher level in tumours than in normal quiescent tissues. © 2001 Cancer Research Campaign http://www.bjcancer.com

Bloom's syndrome is a rare autosomal recessive disorder characterized phenotypically by retarded growth, sun sensitivity, immuno-deficiency and predisposition to a wide variety of cancers at an early age (German, 1993, 1995). This cancer predisposition is of particular interest given that leukaemias, lymphomas and epithelial cancers are all represented. Cells from affected individuals show genetic instability, manifested as an elevated frequency of chromosome breaks and aberrations, as well as the hallmark feature of an approximately 10-fold elevation in the frequency of reciprocal exchanges between sister-chromatids (SCEs) (German, 1993, 1995). The gene mutated in Bloom's syndrome, *BLM*, encodes a member of the RecQ family of DExH box-containing DNA helicases (Ellis et al, 1995). Other RecQ family members include *Saccharomyces cerevisiae* Sgs1p, *Schizosaccharomyces pombe* Rqh1p, and the RECQL, RECQ4, RECQ5 and WRN proteins from human cells (reviewed in Chakraverty and Hickson, 1999; Karow et al, 2000a). Studies using either native or recombinant proteins have indicated that these proteins are helicases that unwind DNA in a 3′–5′ direction (Umezu and Nakayama, 1993; Puranam and Blackshear, 1994; Seki et al, 1994; Gray et al, 1997; Karow et al, 1997; Suzuki et al, 1997; Bennett et al, 1998; Shen et al, 1998). BLM is not the only member of the RecQ family to be mutated in a human genetic disorder. The WRN gene is mutated in the premature ageing disorder, Werner's syndrome (Yu et al, 1996), in which individuals also have an elevated incidence of cancer, particularly sarcomas. Moreover, the RecQ4 gene is mutated in some cases of Rothmund-Thomson syndrome (Kitao et al, 1999), a rare disorder associated with skin and skeletal abnormalities, some features of premature ageing, and a predisposition to certain cancers.

*Received 24 January 2001*
*Revised 13 March 2001*
*Accepted 22 March 2001*

*Correspondence to:* ID Hickson

Cells from Bloom's syndrome individuals show a variety of defects in the maintenance of genome stability. In addition to an elevated frequency of SCEs, Bloom's syndrome cells have a mutator phenotype and an elevated rate of genetic recombination events between homologous sequences, including the formation of quadriradial chromosomes (German, 1993, 1995). Although the precise role of the BLM protein (or indeed of any of the RecQ family helicases) in DNA metabolism remains to be elucidated, many of these proteins appear to play roles in genetic recombination, particularly during DNA replication (Chakraverty and Hickson, 1999). Consistent with such a role, the BLM protein has been shown to interact physically and functionally with replication protein A (RPA) (Brosh et al, 2000), and to bind selectively to the Holliday junction recombination intermediate (Karow et al, 2000b). Indeed, BLM has been shown to promote efficient branch migration of Holliday junctions (Karow et al, 2000b), suggesting a possible role in the production of mature DNA recombinants during repair of DNA damage.

Previous studies using cultured cell lines have indicated that the BLM protein is poorly expressed in non-proliferating cells and peaks during S/G2-phases of the cell division cycle (Dutertre et al, 2000). Moreover, BLM protein has been shown to localize to the nucleus and at certain stages of the cell cycle to be present both in nucleoli and in PML nuclear bodies (Ishov et al, 1999; Zhong et al, 1999; Wu et al, 2000a, 2000b; Yankiwski et al, 2000). However little is known about the expression of the BLM protein in cells and tissues in vivo, or whether BLM is generally expressed in human cancers. Using a specific anti-BLM antibody, which we have validated previously using cell lines and recombinant proteins (Wu et al, 2000a), we have characterized the expression of BLM in normal and neoplastic tissues using immunohistochemistry. We show that BLM is a nuclear protein in human tissues and is expressed at a higher level in proliferating cells of lymphoid origin than in other normal tissues. In contrast, BLM is widely expressed in tumour cells of both lymphoid and epithelial origin.

### MATERIALS AND METHODS

### Antibodies

Generation of the BFL-103 antibody has been described previously (Wu et al, 2000a). The antigen used to raise this mouse monoclonal antibody was full-length recombinant BLM expressed in *Saccharomyces cerevisiae* (Karow et al, 1997). Antibodies specific for proliferating cell nuclear antigen (PCNA; PC10) and Ki67 were obtained from Dako (Denmark).

### Tissues

A range of normal tissues (tonsil, spleen, lymphoid node, thymus, skin, pancreas, testis, colon, kidney, liver, brain and lung) were obtained from the Cellular Pathology Department at the John Radcliffe Hospital, Oxford, UK. Tumour samples from several organ sites were obtained from the frozen tissue bank in the same University Department. The diagnosis was reviewed and confirmed in each case. Cryostat sections of 8 μm were prepared and mounted on poly-L-lysine coated multi-well microscope slides (C. A. Hendley-Essex Ltd, Loughton, Essex, England.) After overnight drying, slides were processed for immunostaining immediately or stored at –20°C until required.

### Immunohistochemistry

Slides were fixed for 10 min in 4% formaldehyde in phosphate-buffered saline. Endogenous peroxidase activity in the samples was blocked for 5 min in Peroxidase Block (Dako K4007) before rinsing in distilled water and washing in PBS. Primary antibodies were then applied to the slides for 90 min at the following concentrations: BFL-103, undiluted tissue culture supernatant from hybridoma cultures; Ki67 (10 μg $ml^{-1}$); PC10 (1 μg $ml^{-1}$). For BFL-103, the secondary antibody used was an Envision Mouse kit (1:200 dilution, Dako K4006). For the Ki67 and PC10 samples, the secondary was a goat anti-mouse HRP conjugate (1:200; Dako PO447). All secondary antibodies were applied for 30 min at room temperature. Slides were counterstained with haematoxylin before mounting in Aquamount (BDH). Substitution of the primary antibody with PBS was used as a negative control

### RESULTS

### Characterization of a BLM-specific monoclonal antibody

The generation of the BFL-103 anti-BLM mouse monoclonal antibody has been described previously (Wu et al, 2000a). BFL-103 was raised in Balb-C mice following injection of purified, recombinant BLM protein. BFL-103 recognizes full-length recombinant BLM using Western blotting, as well as a recombinant fragment representing residues 1–447 of BLM, indicating that the BFL-103 epitope lies in the N-terminal domain of the 1417 amino acid BLM protein. BFL-103 was shown to be specific for BLM by demonstrating a lack of staining of GMO8505 cells isolated from an individual with BS and shown to lack expression of the BLM protein (Wu et al, 2000a).

### Normal tissue distribution of the BLM protein

In all tissues examined, where seen, BLM protein expression was confined to cell nuclei. In normal tissues, no positive staining with the BFL-103 antibody was observed (Figure 1 shows kidney as an example) except in areas where cell proliferation is ongoing. There was high expression in thymic cortex, lymphoid follicles of the tonsil, and around the periarteriolar lymphoid sheath of the spleen. Scattered staining for BLM was also evident in cells from the medulla of the thymus, the cortex and paracortex of the tonsil, the red pulp of the spleen, the placenta, and the basal layers of the skin. These are the areas of cell proliferation in these tissues.

### Expression of the BLM protein in tumour tissues

Positive staining for BLM was observed in tumours of both lymphoid and epithelial origin (Figure 1; Tables 1 and 2). Generally, 5–50% of cells in each tumour sample stained positive for BLM using BFL-103, but in rare cases more than 60% of cells

**Table 1** Summary of staining for BLM in tumour tissues

| Tumour type | No of cases | Nuclear positive (%) | | | |
|---|---|---|---|---|---|
| | | <5 | 5–30 | 30–60 | >60 |
| Breast cancers | | | | | |
| Grade I | 1 | – | 1 | – | – |
| Grade 2 | 3 | – | 3 | – | – |
| Grade 3 | 5 | 1 | 4 | – | – |
| Lymphomas | | | | | |
| Follicular lymphoma | 6 | – | 6 | – | – |
| Large B cell lymphoma | 5 | – | 2 | 3 | – |
| Anaplastic large cell | 4 | – | 3 | 1 | – |
| T cell lymphomas | 5 | – | 3 | 1 | 1 |
| Kidney carcinoma | 5 | 3 | – | 2 | – |
| Lung cancers | | | | | |
| Small cell carcinoma | 4 | – | 1 | 1 | 2 |
| Squamous cell carcinoma | 4 | – | 3 | 1 | – |
| Adenocarcinoma | 4 | – | 3 | 1 | – |
| Colon cancer | 4 | – | 3 | 1 | – |

Tumours are classified both by type and by the percentage of nuclei showing positive staining with BFL-103.

 © 2001 Cancer Research Campaign

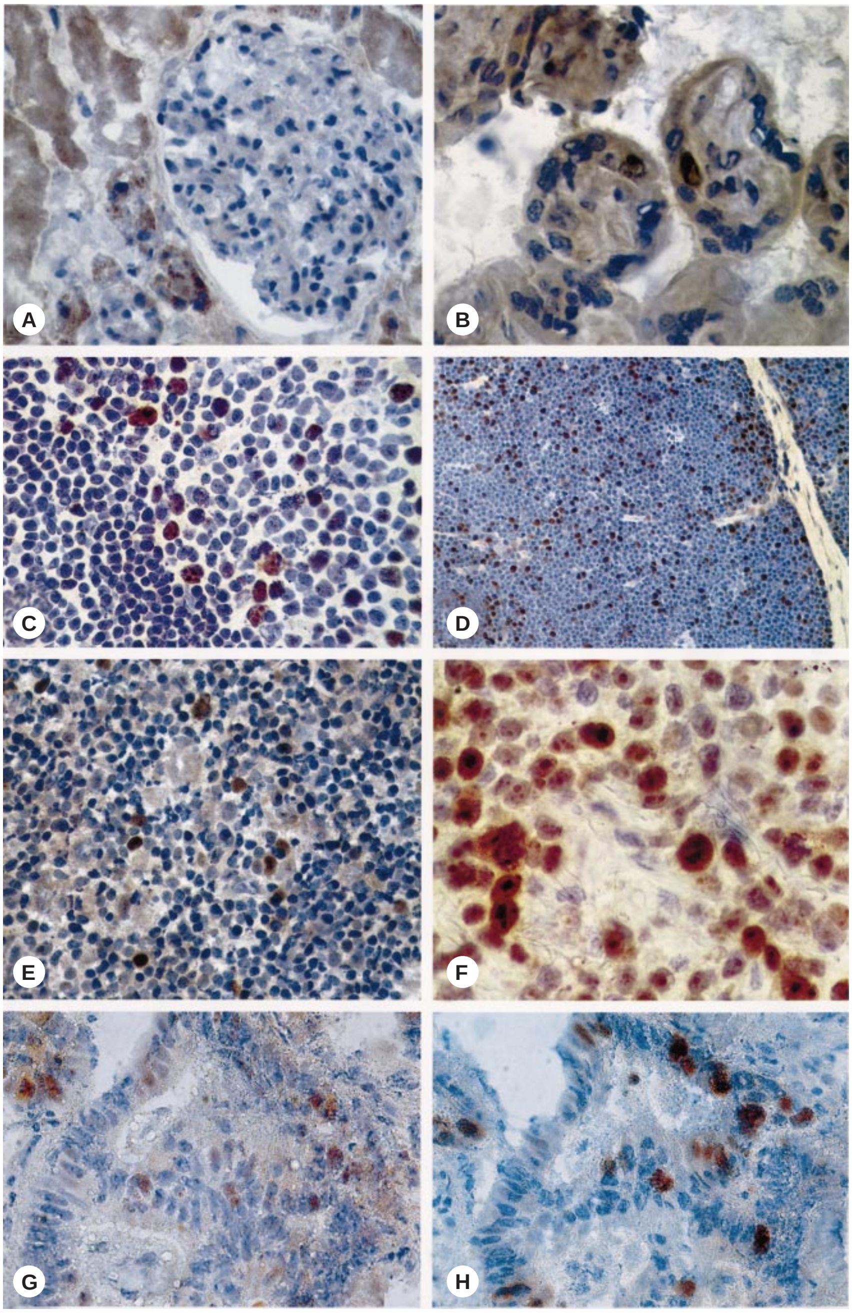

**Figure 1** Staining of human normal and tumour tissues using the anti-BLM specific antibody, BFL-103. Panels **A–D** represent normal tissues, and panels **E–H** represent tumour tissue. Panels: **A**, kidney; **B**, placenta; **C**, tonsil, showing positive nuclei mainly in the germinal centre; **D**, thymus, showing extensive, but scattered positive nuclei; **E**, lymphoma of a large B-cell type; **F**, an example of a breast carcinoma, showing a high level of nuclear staining; **G** and **H** depict the same breast carcinoma sample stained with the BFL-103 and Ki67 antibodies, respectively. Note that a similar percentage of nuclei are stained positive in each case in panels G and H

expressed detectable levels of BLM (Tables 1 and 2). There was a good correlation between the expression of BLM and that of 2 independent, well-established markers of proliferation; PCNA and Ki67 (Table 2). In general, the percentage of cells scoring positive for BLM expression was equal to or a little below the equivalent percentage values for PCNA and Ki67.

### DISCUSSION

We have conducted a detailed analysis of the distribution and expression of the BLM protein in normal and neoplastic human tissues using a specific monoclonal antibody. We have shown that BLM is expressed in only a limited range of normal tissues, such as in lymphoid tissue, and in the skin and digestive tract, with a pattern of expression indicative of BLM being a marker of cellular proliferation. Consistent with this finding, BLM was shown to be widely expressed in tumours of both lymphoid and epithelial origin. In the range of tumours that we studied, the percentage of cells expressing BLM was very similar to the equivalent values for two established proliferation markers, PCNA and Ki67. The slightly lower percentage values for BLM probably reflects the fact that the intensity of staining using the BFL-103 anti-BLM

© 2001 Cancer Research Campaign

**Table 2** Results of staining a variety of neoplastic tissues with BFL 103, compared with staining for two proliferation markers, PC10 (for PCNA) and Ki67

| Tissue | Diagnosis | BFL103 | PC10 | Ki67 |
|---|---|---|---|---|
| Kidney | Renal cell carcinoma | 0 | 0 | 0 |
| Kidney | Renal cell carcinoma | <50 | <50 | <50 |
| Kidney | Renal cell carcinoma | 0 | <1 | <1 |
| Kidney | Seminoma | 50 | 50 | 50 |
| Kidney | Wilms | <1 | <1 | <1 |
| Lung | Small Cell Carcinoma | 40 | 60 | 60 |
| Lung | Small Cell Carcinoma | 70 | 70 | 70 |
| Lung | Small Cell Carcinoma | 70 | 70 | 70 |
| Lung | Small Cell Carcinoma | 30 | 30 | 30 |
| Lung | Squamous Cell Carcinoma | 30–40 | 30 | 30 |
| Lung | Squamous Cell Carcinoma | 30 | 30 | 25 |
| Lung | Squamous Cell Carcinoma | 15 | 15 | 15 |
| Lung | Squamous Cell Carcinoma | 10 | 10 | 10 |
| Lung | Mixed adeno &SQC | 0 | <1 | <1 |
| Lung | Adenocarcinoma | 20 | 20 | 20 |
| Lung | Adenocarcinoma | 30 | 30 | 30 |
| Lung | Adenocarcinoma | 15–20 | 15 | 10 |
| Lung | Papillary Adenocarcinoma | <5 | <5 | <5 |
| Colon | Adenocarcinoma | 35 | 40 | 40 |
| Colon | Adenocarcinoma | 20 | 30 | 30 |
| Colon | Adenocarcinoma | 15 | 20 | 20 |
| Colon | Adenocarcinoma | 20 | 30 | 30 |
| Breast | Adenocarcinoma | 3–5 | 15–20 | 15–20 |
| Breast | Adenocarcinoma | – | – | – |
| Breast | Adenocarcinoma | 3–5 | 5–15 | 5–10 |
| Breast | Adenocarcinoma | 5 | 5 | 5 |
| Breast | Adenocarcinoma | 10 | 25 | 25 |
| Breast | Adenocarcinoma | 5–10 | 15–20 | 15–20 |
| Breast | Adenocarcinoma | 15 | 15–20 | 15–20 |
| Breast | Adenocarcinoma | 10–15 | 10–15 | 10–15 |
| Breast | Adenocarcinoma | 3–7 | 5–10 | 5–10 |
| Lymph node | Follicular Lymphoma | <5 | 5–10 | 5–10 |
| Lymph node | Follicular Lymphoma | 1–5 | 10 | 10–15 |
| Lymph node | Follicular Lymphoma | 5 | 5–10 | 5–10 |
| Lymph node | Follicular Lymphoma | 10–15 | 15–20 | 15–20 |
| Lymph node | Follicular Lymphoma | 5 | 15–20 | 15–20 |
| Lymph node | Follicular Lymphoma | 5 | 5–10 | 5–10 |
| Lymph node | Large B cell lymphoma | 25 | 40 | 40 |
| Lymph node | Large B cell lymphoma | 25 | 35 | 40 |
| Lymph node | Large B cell lymphoma | 15 | 30 | 30 |
| Lymph node | Large B cell lymphoma | 10 | 20–25 | 20–25 |
| Lymph node | Large B cell lymphoma | 5 | 25 | 25 |
| Lymph node | T cell lymphoma | 10–15 weak | 25–30 | 20–25 |
| Lymph node | T cell lymphoma | 15–20 weak | 20 | 15 |
| Lymph node | T cell lymphoma | 10 weak | 22 | 20 |
| Lymph node | T cell lymphoma | | | 40 |
| Lymph node | T cell lymphoma | 5–10 | 25 | 40 |
| Lymph node | T cell lymphoma | 20 | 20–30 | 20–30 |
| Lymph node | T cell lymphoma | 15 | 25 | 25 |
| Lymph node | T cell lymphoma | 35 | 80 | 80 |
| Lymph node | T cell lymphoma | 10 | 15 | 15 |
| Testis | T cell lymphoma | 20 weak | 55 | 45 |

antibody was consistently somewhat weaker than that seen when using the PC-10 or Ki67 antibodies.

BLM is defective in BS, a rare disorder which is associated with several phenotypic abnormalities. Of greatest relevance to the findings of this study, is the fact that BS individuals are cancer prone and, more particularly, succumb at an early age to cancers of all (or nearly all) types, including leukaemias, lymphomas and epithelial tumours (German, 1993, 1995). There is the possibility, therefore, that the *BLM* gene will be defective in a proportion of tumours that arise sporadically in the general population. Although we have not addressed this question directly, it might have been expected that we would have detected loss of BLM staining in some of the tumour samples if BLM expression was lost in a high percentage of any of the tumour types studied. However, we did not obtain any evidence of loss of BLM expression in these tumour samples and therefore we would tentatively conclude that loss of BLM expression does not occur at a high frequency in any of the tumour types studied here. This does not exclude the possibility that mechanisms of BLM inactivation other than loss of BLM protein expression will be found in a significant number of sporadic cancers in humans.

BLM is much more widely expressed in human tumours than in normal tissues, particularly those of epithelial origin. This presumably reflects the far greater fraction of proliferating cells that exists

*© 2001 Cancer Research Campaign*

in tumours than in normal tissues of the same origin. As with other proliferation markers of this sort there is the possibility that BLM might be considered a suitable target for the development of anti-tumour strategies aimed at the elimination of proliferating tumour cells. Moreover, measurement of BLM expression could have some utility in cancer prognosis. Clearly, further work is required to obtain additional evidence that there is any validity to these suggestions.

In summary, we have developed a BLM-specific antibody that is suitable for use in immunohistochemical analysis of human tissues, including primary human tumours. We have shown that the expression of BLM is restricted to the proliferating compartment of normal human tissues, but is widely expressed in tumour tissue of both lymphoid and epithelial origin. BLM is apparently localized exclusively to the nucleus in vivo. With the development of BFL-103, we now have the capability to analyse BLM expression more generally in tumour biopsy material to ascertain whether alterations/abnormalities in BLM expression occur at a measurable frequency in human tumours both before and after antitumour therapy.

### ACKNOWLEDGEMENTS

We thank members of the ICRF Genome Integrity group for valuable discussions, Dr C Norbury for critical reading of the manuscript, and Mrs J Pepper for preparation of the manuscript. Work in the authors' laboratory is supported by the Imperial Cancer Research Fund.

### REFERENCES

Bennett RJ, Sharp JA and Wang JC (1998) Purification and characterization of the Sgs1 DNA helicase activity of *Saccharomyces cerevisiae*. *J Biol Chem* **273**: 9644–9650

Brosh RM Jr, Li JL, Kenny MK, Karow JK, Cooper MP, Kureekattil RP, Hickson ID and Bohr VA (2000) Replication protein A physically interacts with the Bloom's syndrome protein and stimulates its helicase activity. *J Biol Chem* **275**: 23500–23508

Chakraverty RK and Hickson ID (1999) Defending genome integrity during DNA replication: a proposed role for RecQ family helicases. *BioEssays* **21**: 286–294

Dutertre S, Ababou M, Onclercq R, Delic J, Chatton B, Jaulin C and Amor-Gueret M (2000) Cell cycle regulation of the endogenous wild type Bloom's syndrome DNA helicase. *Oncogene* **19**: 2731–2738

Ellis NA, Groden J, Ye TZ, Straughen J, Lennon DJ, Ciocci S, Proytcheva M and German J (1995) The Bloom's Syndrome gene product is homologous to RecQ helicases. *Cell* **83**: 655–666

German J (1993) Bloom Syndrome: A mendelian prototype of somatic mutational disease. *Medicine* **72**: 393–406

German J (1995) Bloom's syndrome. *Dermatol Clin* **13**: 7–18

Gray MD, Shen JC, Kamath-Loeb AS, Blank A, Sopher BL, Martin GM, Oshima J and Loeb LA (1997) The Werner syndrome protein is a DNA helicase. *Nat Genet* **17**: 100–103

Ishov AM, Sotnikov AG, Negorev D, Vladimirova OV, Neff N, Kamitani T, Yeh ETH, Strauss III JF and Maul GC (1999) PML is critical for ND10 formation and recuits the PML-interacting protein Daxx to this nuclear structure when modified by SUMO-1. *J Cell Biol* **147**: 221–233

Karow JK, Chakraverty RK and Hickson ID (1997) The Bloom's syndrome gene product is a 3′–5′ DNA helicase. *J Biol Chem* **272**: 30611–30614

Karow JK, Wu L and Hickson ID (2000a) RecQ family helicases: roles in cancer and aging. *Curr Opin Genet Dev* **10**: 32–38

Karow JK, Constantinou A, Li J-L, West SC and Hickson ID (2000b) The Bloom's syndrome gene product promotes branch migration of holliday junctions. *Proc Natl Acad Sci USA* **97**: 6504–6508

Kitao S, Shimamoto A, Goto M, Miller RW, Smithson WA, Lindor NM and Furuichi Y (1999) Mutations in RECQL4 cause a subset of cases of Rothmund-Thomson syndrome. *Nat Genet* **22**: 82–84

Puranam LL and Blackshear PJ (1994) Cloning and characterisation of RECQL, a potential human homologue of the Escherichia coli DNA helicase RecQ. *J Biol Chem* **269**: 29838–29845

Seki M, Miyazawa H, Tada S, Yanagisawa J, Yamaoka T, Hoshino S, Ozawa K, Eki T, Nogami M, Okumura K, Taguchi H, Hanaoka F and Enomoto T (1994) Molecular cloning of cDNA encoding human DNA helicase Q1 which has homology to Escherichia coli RecQ helicase and localization of the gene at chromosome 12p12. *Nucleic Acids Res* **22**: 4566–4573

Shen JC, Gray MD, Oshima J and Loeb LA (1998) Characterization of Werner syndrome protein DNA helicase activity: directionality, substrate dependence and stimulation by replication protein A. *Nucleic Acids Res* **26**: 2879–2885

Suzuki N, Shimamoto A, Imamura O, Kuromitsu J, Kitao S, Goto M and Furuichi Y (1997) DNA helicase activity in Werner's syndrome gene product synthesized in a baculovirus system. *Nucleic Acids Res* **25**: 2973–2978

Umezu K and Nakayama H (1993) RecQ DNA helicase of Escherichia coli. Characterization of the helix-unwinding activity with emphasis on the effect of single-stranded DNA-binding protein. *J Mol Biol* **230**: 1145–1150

Wu L, Davies SL, North PS, Goulaouic H, Riou JF, Turley H, Gatter KC and Hickson ID (2000a) The Bloom's syndrome gene product interacts with topoisomerase III. *J Biol Chem* **275**: 9636–9644

Wu L, Davies SL and Hickson ID (2000b) Roles of RecQ family helicases in the maintenance of genome stability. *Cold Spring Harbor Symposium of Quantitative Biology* **65**: 573–581

Yankiwski V, Marciniak RA, Guarente L and Neff NF (2000) Nuclear structure in nomal and Bloom syndrome cells. *Proc Natl Acad Sci USA* **97**: 5214–5219

Yu C, Oshima J, Fu Y, Wijsman EM, Hisama F, Alisch R, Matthews S, Najura J, Miki T, Ouais S, Martin GM, Mulligan J and Schellenberg GD (1996) Positional cloning of the Werner's syndrome gene. *Science* **272**: 258–262

Zhong S, Hu P, Ye T-Z, Stan R, Ellis NA and Pandolfi PP (1999) A role for PML and the nuclear body in genomic stability. *Oncogene* **18**: 7941–7947

*© 2001 Cancer Research Campaign*
